# Click for Care, Care for Planet: Sustainable Marketing Drivers of Telemedicine Adoption

**DOI:** 10.1177/26924366251394120

**Published:** 2025-10-31

**Authors:** Altuğ Ocak

**Affiliations:** Department of Travel, Tourism and Entertainment Services, Istanbul Beykent University, Istanbul, Turkiye.

**Keywords:** telemedicine, sustainable marketing, environmental benefit, trust, attitude, behavioral intention

## Abstract

**Purpose:**

This study examines telemedicine as a sustainable service innovation that combines health, environmental and convenience benefits. It explores how these perceived benefits influence attitudes and intentions to use telemedicine and how trust in the provider shapes this relationship.

**Design/Methodology:**

A cross-sectional online survey was conducted with 400 adults who had used or planned to use telemedicine. Data were analyzed using partial least squares structural equation modeling with 5,000 bootstraps.

**Findings:**

Perceived environmental (β = 0.318, *p* < 0.001), health (β = 0.267, *p* < 0.001) and convenience benefits (β = 0.231, *p* < 0.001) all positively affected attitudes toward telemedicine. Attitude, in turn, predicted behavioral intention (β = 0.412, *p* < 0.001). Trust in provider strengthened the link between attitude and intention (β = 0.073, p = 0.002). The model explained 69% of variance in attitude and 74% in intention (*R*^2^ = 0.69; *R*^2^ = 0.74).

**Practical Implications:**

Results show that emphasizing both health and environmental value can enhance public acceptance of telemedicine.

**Originality/Value:**

By integrating sustainable marketing logic with the Theory of Planned Behavior, this study highlights telemedicine’s role in advancing eco-efficient, patient-centered health care adoption.

## Introduction

Telemedicine has become a mainstream model of care, no longer just a contingency plan. In addition to access and continuity advantages, virtual visits avoid or minimize patient and clinician travel, facility utilization, and can decrease the environmental impact of care processes. Positioned within the sustainable marketing paradigm, telemedicine thus presents an attractive double-value proposition: improved access to and outcomes of health and a reduced environmental footprint (e.g., travel and emissions saved), with the convenience that service consumers all value. However, whereas e-health adoption research routinely prioritizes perceived usefulness, ease, and risk, relatively less is understood regarding how environmental co-benefits need to be communicated and influence attitudes and intentions compared to health and convenience benefits. Similarly, TR, salient in e-health settings due to privacy and safety considerations, can condition whether positive judgments of telemedicine are realized as actual BIs.

Based on the Theory of Planned Behavior and sustainability marketing, this research formulates and empirically examines an integrative framework within which perceived environmental benefit (PEB), perceived health benefit (PHB), and perceived convenience (PC) collectively influence attitude toward telemedicine (ATT) and behavioral intention (BI). We also suppose that trust in provider (TR) reinforces attitude to intention conversion (moderation) and ATT carries the impacts of PEB, PHB, and PC to BI (mediation). We test the model empirically with a cross-sectional survey and variance-based partial least squares structural equation modeling (PLS-SEM) with bootstrapping.

### Research problem and questions

In spite of increasing interest in eco-friendly services, there are three gaps. One, the position of environmental value in health care service adoption is under-specified: Do customers consider sustainability in their telemedicine decision, and to what extent in relation to health and convenience? Two, the process through which such benefit perceptions cause intention, through attitude, is seldom examined in a composite model that provides for both indirect and direct effects. Third, the boundary condition of trust, which is frequently crucial in e-health, has not been tested systematically as an attitude-intention relation strengthener in the sustainability setting.

So we ask:

1. How do PEB, PHB, and PC affect ATT and BI?
2. Does ATT mediate the effect of these benefits on BI?
3. Does TR enhance the impact of ATT on BI?

Study Objectives are:

• PEB, PHB, and PC have a direct impact on ATT and BI.
• Investigate the mediating effect of ATT between each benefit (PEB, PHB, PC) and BI.
• Examine the moderating effect of TR on the ATT → BI relation.
• Assess explanatory power (*R*^2^) of ATT and BI within a comprehensive sustainable-marketing framework for telemedicine.

### Overview of the proposed model and hypotheses

The theory suggests positive influence of PEB, PHB and PC on ATT (H1-H3); a positive influence of ATT on BI (H4); additive direct influences of PEB, PHB and PC on BI (H5-H7); positive moderation of TR on ATT → BI (H8); and mediation of PEB/PHB/PC through ATT to BI (H9-H11). The study is planned to capture partial mediation with the potential for instrumental beliefs (health, convenience, sustainability) to directly influence intention.

### Contributions

This research offers four contributions:

1. Theory: It connects sustainability value with digital health adoption and demonstrates that environmental benefit is an essential driver, rather than a secondary trait, of telemedicine adoption.
2. Mechanism: It explains attitude’s mediating function in converting many benefit perceptions to intention with latitude for residual direct effects.
3. Boundary condition: It makes provider trust a condition that enhances the attitude-intention relationship in telemedicine.
4. Practice and policy: It offers practical advice on messaging (health-first, environment-reinforced), trust signaling (privacy/security transparency), and impact disclosure (e.g., CO_2_ saved), synchronized with telemedicine marketing with sustainability objectives.

### Structure of the article

Section “Theoretical Background and Hypotheses” formulates the theoretical background and hypotheses. Section “Methods” outlines the methodology. Section “Results” presents the results (measurement, structural paths, *R*^2^, mediation, and moderation). Section “Discussion and Implications” covers discussion of contributions, implications for managers and policymakers, limitations, and directions for future research.

## Theoretical Background and Hypotheses

### Conceptual background

This research investigates telemedicine as a long-term sustainable service innovation with a value proposition for health outcomes, reduced environmental effects (e.g., travel and emissions avoided) and service convenience. The Theory of Planned Behavior (TPB) suggests that attitude is the proximal antecedent to intention, based on salient consequence beliefs.^1^ We synthesize sustainability marketing research that positions ecological and social value as desirable market outcomes and electronic-health adoption research that highlights trust and service quality in e-health uptake.^2–4^ Convenience is consistently ranked as a key driver of positive evaluations and decision-making in service contexts.^5^

In recent years (2022–2025), increasing empirical work has analyzed environmental, social and economic aspects of telemedicine adoption, in line with revived interest in sustainable health care provision. For example, a recent life-cycle analysis estimated emissions and energy savings for televisit models, confirming telemedicine’s carbon-mitigating potential.^6^ Other studies investigated patient beliefs about telemedicine with overt consideration of eco-consciousness, demonstrating that payers for greener care options have stronger adoption intentions. Correspondingly, some comparative cross-country studies have begun to capture how regulatory systems, digital infrastructure and cultural beliefs shape sustainable telehealth adoption across nations. Methodologically, recent work juxtaposed qualitative interviews with quantitative environmental impacts models, diversified indicators of sustainability considered beyond travel-based emissions (e.g., facility energy, materialization) and added life-cycle and circular-economy perspectives. Despite establishing an empirical base, their focus on environmental co-benefits as primary motivators remains rudimentary, which highlights a need for theory-informed work like the current study to systematically combine sustainability, health and convenience beliefs across telemedicine adoption models.

### Sustainability and green health care

Sustainability in health care moves even beyond outcomes of clinical care to environmental, social and economic aspects of health care delivery. Shifting health care towards green health care aims to reconcile service efficiency and ecological responsibility, lessening environmental impacts hitherto inherently linked to face-to-face medical visits. Here, telemedicine becomes a key driver of sustainable health systems by its ability to reduce travel, resource utilization and facility-based emissions.

More recent studies, in Environmental Health Perspectives, performed an integrated qualitative and life-cycle evaluation revealing that televisit procedures significantly lower carbon emission as well as energy usage for face-to-face consultation activities, yet enhance patient accessibility as well.^6^ Their studies emphasize that telemedicine is capable of supporting decarbonization measures in health care when properly utilized. Likewise, another study, published in Journal of Environmental Psychology, identify how pro-environmental behavior by health care consumers and professionals, e.g., preference for low-carbon service options, finds its driving force based on moral identity and environmental concern.^7^ Such behavior determinants indicate how environmental awareness is capable of not just influencing clinical decisions, but also information technology adoption in health care.

Including a framework for decision-making, recent research finds doctors and patients are becoming more inclined to carbon-footprint consideration in ethical clinical decision-making processes.^8^ The research outcomes were that health care-associated emission awareness influences organizational policy and also patient attitude, revealing environmental cues are now having their influence on physician behavior. Overall, these pieces of research position telemedicine as part of a broader trend to sustainable and responsible health care, a point of relevance being environmental co-benefits are key, not secondary, drivers of perceived value for digital health technologies.

Therefore, our study extends these findings by investigating how perceived environmental advantages, combined health and convenience beliefs, mold patient attitudes BIs regarding telemedicine as a sustainable service innovation.

### Sustainable marketing in digital health care

Sustainable marketing places environmental co-benefit messaging alongside primary service utility.^2,9^ For telemedicine, sustainability derives mainly from avoided patient and clinician travel and resource-conserving visits, which can be signaled credibly to consumers as PEB. Telemedicine also provides health benefits (improved access, continuity of care, reduced exposure) and convenience (time savings, easy scheduling). Because consumers evaluate services holistically, sustainability messages may complement rather than replace health and convenience framing.

### Digital health adoption and trust mechanisms

Though the TPB offers a strong foundation for predicting technology acceptance based on attitudes, subjective norms and perceived behavioral control, current studies of digital health adoption stress a need for expanding traditional models by including contextual and trust factors. Developments in health care technologies have called for a new knowhow of consumer decisions based on a state of digital uncertainty, a condition of sensitivity related to information privacy and mediating care across platforms.

More recently, a version of the Unified Theory of Acceptance and Use of Technology modified the model for health care applications by incorporating patient empowerment, health anxiety and data transparency as central adoption drivers.^9^ As a complement, a recent analysis of trust mechanisms for digital health platforms found platform reliability, provider credibility and assurances of privacy to be central antecedents of continued use.^10^ Their work highlights how trust serves both a belief-based and a relational function, acting to mediate between technological interface and perceived provider integrity an important connection for health care service exchanges, where data exchange is of a sensitive nature and physical contact is limited.

From a consumer psychology point of view, one study conducted recently analyzed the paradox of privacy of telemedicine, observing how consumers report a great deal of concern regarding privacy, yet also actively utilize digital health platforms by reasons of convenience, accessibility, and perceived need.^11^ Their work identifies a cognitive dissonance between perceived risk of privacy and intention behavior, but trust helps alleviate it by bringing about a sense of protection and management of data processing procedures.

Incorporating these outcomes fortifies applicability of our current model to digital health by positioning TR not merely as a moderating process, but also as a principal relational process between positive attitudes and BI. Both TPB and existing digital adoption models of current health care systems encompass cognitive-evaluative as well as relational-trust processes yielding sustainable adoption of telemedicine across existing health care systems.

### Sustainable service design in health care

Sustainable service development focuses on incorporating ecological and social factors into health care delivery’s intrinsic framework. Telemedicine, aside from its clinical efficiency, can be framed as a sustainable service development aligned with circular economies, life-cycle efficiency, and long-term health systems innovation diffusion.

In a recent article, it was contended that the principles of circular economies, maximizing waste reduction, elongating resource usefulness, and designing for recyclability, could be extended to health care services.^12^ Telemedicine also complies with a similar rationale by diminishing physical resource requirements, utilizing digital platforms as reusable infrastructure, and diminishing carbon-intensive facilities utilization.

From an evaluative perspective, a recent study demonstrated that telehealth interventions yield measurable reductions in greenhouse gas emissions, energy consumption, and material waste across their entire life cycle.^13^ These findings provide empirical evidence that telemedicine is not only a convenient substitute for in-person care but also an environmentally efficient design that contributes to sustainable health system transitions.

Innovation diffusion extent, a new study examined how sustainable service innovations spread across health care systems, finding their adoption to be a function of both value congruence and technological readiness of sustainability-oriented policies and cultural environments, respectively.^14^ With its environmental and convenience advantages, it is then possible for telemedicine’s prospects for diffusion as a dominant sustainable health service to be improved significantly.

They indicate together how telemedicine represents more than a virtual device, but a part of a larger trend toward paradigms balancing resource optimization, sustainability-driven innovation, and patient outcomes.

### Constructs and definitions

PEB is the perception that using telemedicine reduces environmental degradation such as emissions and resource wastage.^2,15,16^ PHB refers to the belief that telemedicine enhances one’s health care by improving access, continuity, outcomes and reducing exposure.^17,18^ PC is the perception of time savings, scheduling flexibility, and ease of use.^5^ ATT is the overall positive evaluation of using telemedicine.^1^ TR is the expectation that providers offering telemedicine are competent, benevolent, and safeguard privacy.^3^ BI is the self-reported likelihood of using or recommending telemedicine.^19^

### Hypotheses development

#### Antecedents of attitude

In the TPB, attitude is a judgment of a behavior that is both positive or negative and is a function of salient beliefs about consequential expectations.^1^ In the case of telemedicine, three belief categories are particularly salient, PEB, PHB PC, and each represents a unique value creation dimension and therefore can contribute to ATT in a positive manner.

#### Perceived environmental benefit

Sustainability marketing suggests that consumers increasingly make service choices based on environmental values.^2,7^ Telemedicine significantly reduces clinician and patient travel and limits energy-intensive use of facilities, thereby decreasing greenhouse-gas emissions and other environmental impacts.^20^ When consumers perceive such benefits, they tend to experience moral satisfaction and identity congruence, which foster favorable attitudes.^21^ Thus, environmental benefit can be a significant attitudinal motivator, even in a traditionally clinically dominated health care setting.

#### Perceived health benefit

Health beliefs are central determinants of preventive and service-seeking behavior.^17^ Telemedicine assures timely access to care, continuity for chronic disease, and decreased exposure to infectious illness.^2^ These impacts directly improve feelings of individual and family well-being, which in turn foster a more positive overall assessment of telemedicine.

#### Perceived convenience

Convenience is a strong driver of service quality across industries.^5^ Web-based health services offer on-demand scheduling, eliminate waiting and travel time, and provide multi-device access, all of which reduce the cognitive and temporal costs of obtaining care.^22,23^ Such efficiency gains are likely to elicit positive affect and perceived control, thereby strengthening favorable attitudes. Together, the three belief systems, sustainability (environmental) value, functional core value (health), and experience value (convenience), provide comprehensive motivation. Consistent with the TPB and empirical evidence in green services and e-health, all are expected to significantly influence ATT.^24,25^

H1: Perceived Environmental Benefit positively influences Attitude toward Telemedicine.

H2: Perceived Health Benefit positively influences Attitude toward Telemedicine.

H3: Perceived Convenience positively influences Attitude toward Telemedicine.

#### Attitude and BI

In the TPB, attitude is the most proximal cognitive predictor of BI, which represents an individual’s overall positive or negative evaluation of performing a target behavior.^1^ As consumers recognize that a service is useful, convenient, and aligned with their personal or moral values, they are more likely to form positive attitudes, which in turn incline them to act. Previous studies in digital health and sustainable services confirm that attitude is a key conduit through which benefit perceptions are translated into adoption intention.^19,26,27^ For telemedicine, positive attitudes can develop because of clinical assurance (better access and outcomes), sustainability benefits (reduced travel and emissions), and practical convenience (time saved, flexible scheduling). Such favorable evaluations reduce perceived behavioral barriers and enhance confidence that telemedicine is an attractive and satisfactory option.^22,28^ Sustainability research further shows that when health services are presented as environmentally responsible, individuals may feel moral satisfaction and identity congruence, which also fosters positive attitudes and intention.^24^ Therefore, we hypothesize that ATT positively influences BI to use telemedicine.

H4: Attitude toward Telemedicine positively influences Behavioral Intention to use telemedicine.

#### Direct effects of PEB, PHB, and PC on BI

While the TPB positions attitude as the strongest predictor of BI, salient and personally relevant beliefs can also exert direct effects on intention.^1,19^ In digital health and green services, these direct paths complement attitudinal mediation, often yielding partial mediation patterns documented in PLS-SEM.^29^

#### Perceived environmental benefit

Environmental consciousness is increasing, and many individuals adopt low-carbon services to express ecological identity and reduce personal carbon footprints, even when their lifestyles are only moderately green.^2,16^ In telemedicine, recognition of avoided travel and emissions can therefore trigger an immediate, value-based intention to use the service as a pro-environmental action.

#### Perceived health benefit

Health motivations are typically instrumental and urgent, especially for people with chronic illness or elevated disease risk. Research on mobile health adoption shows that expectations of improved health outcomes can motivate use independently of attitudinal assessments.^18,27^ PHB is thus expected to have a direct positive effect on BI.

#### Perceived convenience

Convenience, time saving, scheduling flexibility, and reduced effort have long been direct drivers of technology and service uptake.^17,18^ The practical advantages of telemedicine, such as eliminating travel and waiting times, can lead busy consumers directly to adopt the service without necessarily shaping attitude.

Based on these premises, we argue that each of the three benefit perceptions exerts both direct and indirect (attitude-mediated) effects on behavioral intention.

H5: Perceived Environmental Benefit positively influences Behavioral Intention to use telemedicine.

H6: Perceived Health Benefit positively influences Behavioral Intention to use telemedicine.

H7: Perceived Convenience positively influences Behavioral Intention to use telemedicine.

#### Moderating role of trust in provider

Trust is a key factor in mitigating perceived risk in electronic health environments, such as concerns over data privacy, misdiagnosis, and lack of physical examination. Trust reflects the expectation that providers are competent, benevolent, and protective of confidential information.^3^ When trust is high, consumers are more willing to act on their positive evaluations of a service. This perspective is supported by relationship marketing theory, which treats trust as relational capital that converts attitudes into behaviors, and by technology adoption research identifying trust as a critical determinant of online service acceptance.^6,26,27^

Telemedicine involves the transfer of confidential health data and real-time interactions with clinicians who may be unfamiliar to patients. These circumstances can heighten perceived risk (privacy violations, clinical quality) and create psychological distance, potentially weakening the attitude—intention link.^22^ Trust counteracts these effects by enhancing credibility and reducing uncertainty, thereby enabling positive attitudes, formed from environmental, health, and convenience advantages, to more strongly predict the intention to use telemedicine.^30^ Accordingly, we hypothesize that trust acts less as an independent motivator and more as a boundary condition, strengthening the influence of attitude on BI when provider competence and integrity are assured.

H8: Trust in Provider strengthens the positive relationship between Attitude toward Telemedicine and Behavioral Intention to use telemedicine.

#### Mediating role of attitude

In the TPB, attitude toward a behavior is the core cognitive mechanism that converts salient beliefs into BI.^1^ Consumers weigh anticipated outcomes, such as environmental, health, and convenience gains, and these evaluations crystallize into an overall attitude, which then drives intention. This indirect process has been repeatedly demonstrated in sustainable consumption, e-health adoption, and internet service utilization.^24,29^

##### Perceived Environmental Benefit

Individuals experience environmental identity congruence and moral satisfaction when they believe telemedicine reduces resource use and emissions, which in turn fosters favorable attitudes and increases intention.^2,21^

##### Perceived Health Benefit

PHBs, enhanced access, care continuity, and reduced infection risk form a core incentive for telemedicine. In line with the Health Belief Model, such benefits cultivate positive attitudes that drive adoption.^17,18^

##### Perceived Convenience

Convenience, reflected in time savings, scheduling flexibility, and effort reduction, is well established in service marketing literature as a determinant of overall service evaluations that enhance usage intention.^5,22^

TPB also allows for partial mediation, in which benefit perceptions can influence intention indirectly (through attitude) and directly when they are highly salient or diagnostic.^1,20^ Accordingly, we hypothesize that ATT mediates the relationships between each of the three benefit perceptions and BI.

H9: Attitude toward Telemedicine mediates the relationship between Perceived Environmental Benefit and Behavioral Intention.

H10: Attitude toward Telemedicine mediates the relationship between Perceived Health Benefit and Behavioral Intention.

H11: Attitude toward Telemedicine mediates the relationship between Perceived Convenience and Behavioral Intention.

### Proposed research model

**FIG. 1.**
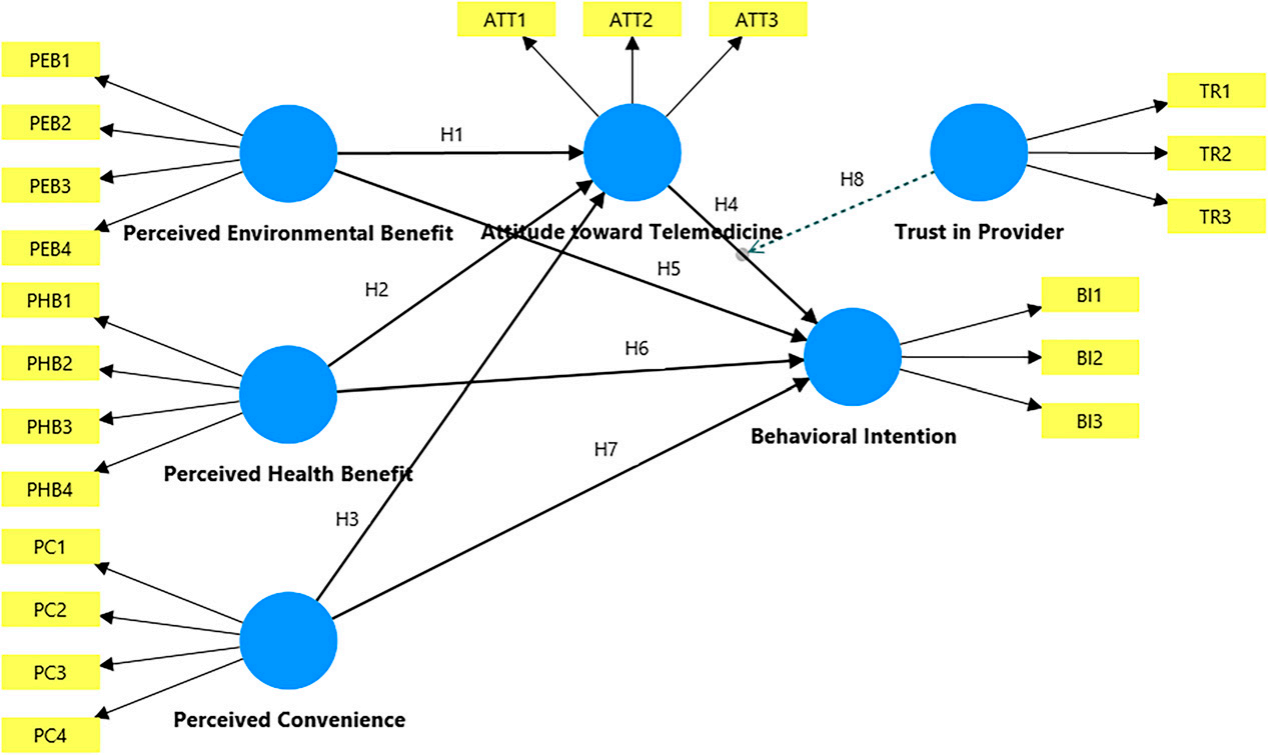
Proposed research model showing hypothesized relationships.

Figure 1 presents the conceptual model that synthesizes the theorized relations between the PEB, PHB, PC, ATT, TR, and BI constructs.

## Methods

### Research design and context

We utilized quantitative, cross-sectional survey to investigate ATT and BI antecedents and analyze TR moderation and ATT mediation. Partial least squares PLS-SEM was used as it is ideally compatible with prediction models, complexity paths (moderation + mediation) and distributional robustness.^31^

### Sampling, power and data collection

The population of interest comprised adult consumers who had either used telemedicine within the past 12 months or were likely to do so in the upcoming 12 months. We applied purposive sampling through mailing lists of health care providers and social media groups. *A priori* power analysis using G*Power with α = 0.05 and power = 0.80 for a five-predictor multiple-regression model with a small effect size (f^2^ = 0.02) indicated a minimum sample of ∼395 respondents. We obtained ∼400 completed surveys, meeting both the power requirement and the PLS “10× rule”.^31,32^ Data were collected online over a four-week period. Pilot testing (*n* = 30) ensured clarity of items and appropriate survey length.

### Measures and Instrument Development

All constructs were measured with multi-item, 5-point Likert scales (1 = strongly disagree, 5 = strongly agree). Items were adapted from validated literature for the telemedicine context:

• PEB, capturing eco-impact and travel/CO_2_ reduction;^7^
• PHB, covering access, continuity, and exposure reduction, based on health-belief and telemedicine research;^4,17^
• PC, reflecting time savings, scheduling flexibility, and ease of use;^5^
• ATT, assessing overall positive evaluation of telemedicine use;^1^
• TR, reflecting perceptions of provider competence, benevolence, and privacy/security protection;^3^ and
• BI, indicating intention, recommendation, and preference for telemedicine.^19^

Where necessary, the instrument was translated and back-translated to ensure conceptual equivalence.^33^ Content validity was confirmed by a panel of marketing and health-services scholars and two clinicians. The final questionnaire included screening questions, an attention-check item, a brief history of prior telemedicine use, and demographic items.

### Procedure, data screening and missingness

Participation was voluntary and anonymous. Responses failing attention checks or showing straight-lining were removed. Missing data were <5% at item level and handled via pairwise deletion.^31^ Descriptives and normality diagnostics were inspected prior to modeling.

### Common method bias and non-response bias

To minimize common method bias (CMB), procedural remedies were implemented, including assuring respondent anonymity, randomizing item order, and using heterogeneous response anchors. Ex-post diagnostics were also conducted. Harman’s single-factor test indicated that a single factor accounted for <50% of the variance and full collinearity variance inflation factors (VIFs) were all below conservative cut-offs, suggesting that CMB is unlikely to bias the estimates.^1,34^ Non-response bias was assessed by comparing early and late respondents, with no significant differences found.^35^

### PLS-SEM estimation and evaluation strategy

Data were processed with SmartPLS 4 and partial least squares PLS-SEM based on 5,000 bootstrap resamples (two-tailed) in order to assess the significance of paths and indirect effects. As suggested by the two-step approach, we estimated the measurement model first by looking at indicator reliability (outer loadings ≥0.70), internal consistency (Cronbach’s α and composite reliability ≥0.70), and convergent validity (average variance extracted (AVE) ≥0.50).^31^ To check for discriminant validity, we employed the Fornell–Larcker criterion (square root of AVE higher than inter-construct correlations) and heterotrait–monotrait ratio (HTMT <0.90).^36^ Collinearity of construct and indicator was assessed using VIF (<5). The structural model was then analyzed in the second step for importance and significance of paths with the aid of bootstrapped t-values and confidence intervals (CIs) and for explanatory power using the coefficient of determination (*R*^2^), effect size (f^2^), and predictive relevance (Q^2^) generated through blindfolding. Model fit at the model level was also defined in terms of standardized root mean square residual (SRMR), normed fit index (NFI), and discrepancy measures d_ULS and d_G. The estimation and assessment approach overall framed a stringent test of the proposed effects, such as the moderating role of TR and the mediating role of ATT.

### Moderation specification (TR)

Moderating effect of TR on ATT → BI (H8) was latent-variable defined with the product-indicator approach following PLS-SEM best practice.^1,31,36^ In our definition, ATT×TR interaction served as a product composite (single indicator in the measurement outputs); significance was tested through bootstrapped interaction paths. For interpretability, we explored the conditional effect of ATT on BI at ±1 standard deviation (SD) of TR.

### Mediation testing

Mediation effects of ATT on PEB/PHB/PC to BI (H9-H11) were tested through bootstrapped indirect effects with percentile 95% CIs; mediation is confirmed when indirect effect is significant (CI not including zero). We present direct, indirect, total effects, and variance accounted for (VAF) in order to categorize mediation as partial versus full.^37,38^

### Ethics

This study involved an anonymous, voluntary online survey of adults and collected no identifiable personal health information. In accordance with the Declaration of Helsinki, formal institutional review board/ethics committee approval was not requested for this minimal-risk research. Participants were informed of the study purpose and data use; consent was implied by completion of the survey.

## Results

Analyses proceeded with the suggested two-step PLS-SEM procedure: (1) test measurement model (reliability/validity) and (2) test structural model (paths, *R*^2^, mediation).

### Sample characteristics

The study also surveyed a total of 400 adult respondents. The respondents sample comprised 52.5% females and 47.5% males, representing a good, even division between both genders for a well-balanced sample set. Respondents mean age was 38.4 years (SD = 10.6) and respondents ranged across an age spectrum of 20–65 years of age. On their educational background, 62% of respondents possessed a university degree, 25% possessed postgraduate studies and 13% possessed a high school/vocational certification, presenting a highly educated and digitally literate set of respondents typical of user groups for standard telemedicine services.

With regard to familiarity with telemedicine, 71% reported a minimum of one prior consultation of telemedicine in the past 12 months, whereas 29% indicated an intention to use telemedicine services in the following year. This combination ensured covering both current and prospective users, hence a realistic assessment of BI formation.

Recruitment of respondents happened by purposive sampling of health provider email lists and health-based internet social media groups. It required four weeks for data collection, hence controlling for temporal variation bias by having one-time surveys. The demographic scope adds to generalizability of findings to adult populations actively engaging in digital space and growing exposure to telehealth services.

### Global model fit

Total fit indices show a great fit for the model to be estimated: SRMR = 0.025, NFI = 0.937, d_ULS = 0.149, d_G = 0.234 (saturated vs. estimated nearly identical), with anticipated sample-size sensitivity of χ^2^. These indices establish adequacy of the model.

### Measurement model

#### Reliability and convergent validity

All measures load strongly on respective constructs and are highly significant (two-tailed bootstrap). Composite reliability and AVE are substantially above general cutoffs (see Table 1).

**Table 1.** Outer Loadings, Original, Mean, SD, t, and *p*

Construct → indicator	O	M	SD	t	*p*
Attitude toward telemedicine (ATT)					
ATT1	0.943	0.943	0.006	146.084	<0.001
ATT2	0.939	0.939	0.007	144.360	<0.001
ATT3	0.947	0.947	0.006	160.841	<0.001
Behavioral intention (BI)					
BI1	0.937	0.937	0.007	134.659	<0.001
BI2	0.945	0.945	0.006	158.260	<0.001
BI3	0.951	0.951	0.005	178.437	<0.001
Perceived convenience (PC)					
PC1	0.916	0.916	0.008	108.296	<0.001
PC2	0.910	0.909	0.009	98.973	<0.001
PC3	0.909	0.909	0.009	98.471	<0.001
PC4	0.911	0.911	0.009	101.504	<0.001
Perceived environmental benefit (PEB)					
PEB1	0.919	0.919	0.008	110.343	<0.001
PEB2	0.917	0.917	0.009	107.297	<0.001
PEB3	0.931	0.931	0.007	134.277	<0.001
PEB4	0.916	0.915	0.008	112.067	<0.001
Perceived health benefit (PHB)					
PHB1	0.919	0.918	0.008	115.685	<0.001
PHB2	0.922	0.922	0.008	119.935	<0.001
PHB3	0.919	0.919	0.008	115.484	<0.001
PHB4	0.926	0.927	0.007	131.623	<0.001
Trust in provider (TR)					
TR1	0.948	0.948	0.006	157.878	<0.001
TR2	0.935	0.935	0.008	123.204	<0.001
TR3	0.951	0.951	0.005	188.010	<0.001

M, mean; O, original; SD, standard deviation.

All loadings ≥0.909 (*p* < 0.001). AVE ≥0.831 and CR ≥0.952 substantiate convergent validity and internal consistency (see Table 2).

**Table 2.** Construct Reliability and Validity

Construct	Cronbach’s α	ρ_a_	ρc	AVE
ATT	0.938	0.938	0.960	0.889
BI	0.939	0.939	0.961	0.892
PC	0.932	0.932	0.952	0.831
PEB	0.940	0.941	0.957	0.848
PHB	0.941	0.941	0.957	0.849
TR	0.940	0.944	0.961	0.892

AVE, average variance extracted.

#### Indicator collinearity

All outer VIFs are <5.0 (range 1.000–4.735), indicating no severe collinearity. Retention is justified given strong loadings/content (see Table 3).

**Table 3.** Collinearity Statistics (Outer-Model VIFs)

Construct	Item	VIF
ATT	ATT1 4.158	ATT2 3.912
BI	BI1 3.843	BI2 4.347
PC	PC1 3.596	PC2 3.377
PEB	PEB1 3.741	PEB2 3.712
PHB	PHB1 3.694	PHB2 3.806
TR	TR1 4.648	TR2 3.861

VIFs, variance inflation factors.

#### Discriminant validity (HTMT)

All HTMT values <0.90, supporting discriminant validity (largest = ATT-BI = 0.892, within the <0.90 criterion) (see Table 4).

**Table 4.** HTMT Matrix

Construct	ATT	BI	PC	PEB	PHB	TR	ATT×TR
ATT		0.892	0.725	0.750	0.779	0.437	0.128
BI			0.731	0.726	0.785	0.468	0.200
PC				0.561	0.554	0.401	0.107
PEB					0.615	0.412	0.090
PHB						0.456	0.105
TR							0.156

HTMT, heterotrait–monotrait ratio.

### Structural model (hypotheses testing)

Bootstrapping (5,000 resamples; two-tailed) also supports all theorized effects (see Table 5).

**Table 5.** Structural Path Coefficients

Path	β (O)	M	SD	t	p	Support
H1: PEB → ATT	0.318	0.318	0.034	9.465	<.001	Yes
H2: PHB → ATT	0.387	0.387	0.036	10.642	<.001	Yes
H3: PC → ATT	0.310	0.310	0.034	9.126	<.001	Yes
H4: ATT → BI	0.425	0.424	0.047	9.133	<.001	Yes
H5: PEB → BI	0.132	0.133	0.033	3.994	<.001	Yes
H6: PHB → BI	0.232	0.232	0.035	6.694	<.001	Yes
H7: PC → BI	0.186	0.186	0.033	5.687	<.001	Yes
H8: ATT × TR → BI	0.073	0.073	0.023	3.156	.002	Yes

SD, standard deviation

### Coefficient of determination (*R*^2^)

The model explains 69% of the variance in ATT and 74% in BI, indicating strong explanatory power (see Table 6).

**Table 6.** *R*^2^ of Endogenous Constructs

Endogenous	Predictors	*R*²	Interpretation
ATT	PEB, PHB, PC	0.69	Substantial
BI	ATT, PEB, PHB, PC, ATT×TR	0.74	Substantial

### Mediation analysis

Indirect effects via ATT are significant and positive for all three antecedents, indicating partial mediation (direct paths remain significant) (see Table 7).

**Table 7.** Total Indirect Effects via ATT (Bootstrapped)

Indirect path	Indirect β (O)	M	SD	t	p	Supported
H9: PEB → ATT → BI	0.135	0.135	0.022	6.180	<0.001	Yes
H10: PHB → ATT → BI	0.164	0.164	0.024	6.881	<0.001	Yes
H11: PC → ATT → BI	0.132	0.131	0.019	6.845	<0.001	Yes

SD, standard deviation

All indirect are significant (*p* < 0.001). VAF values around 41–51% fall in the partial mediation range (see Table 8).

**Table 8.** Effect Decomposition and VAF

Predictor → BI	Direct β	Indirect β (via ATT)	Total effect	VAF (=indirect/total)	Mediation
PEB	0.132	0.135	0.267	50.6%	Partial
PHB	0.232	0.164	0.396	41.4%	Partial
PC	0.186	0.132	0.318	41.5%	Partial

VAF, variance accounted for.

**FIG. 2.**
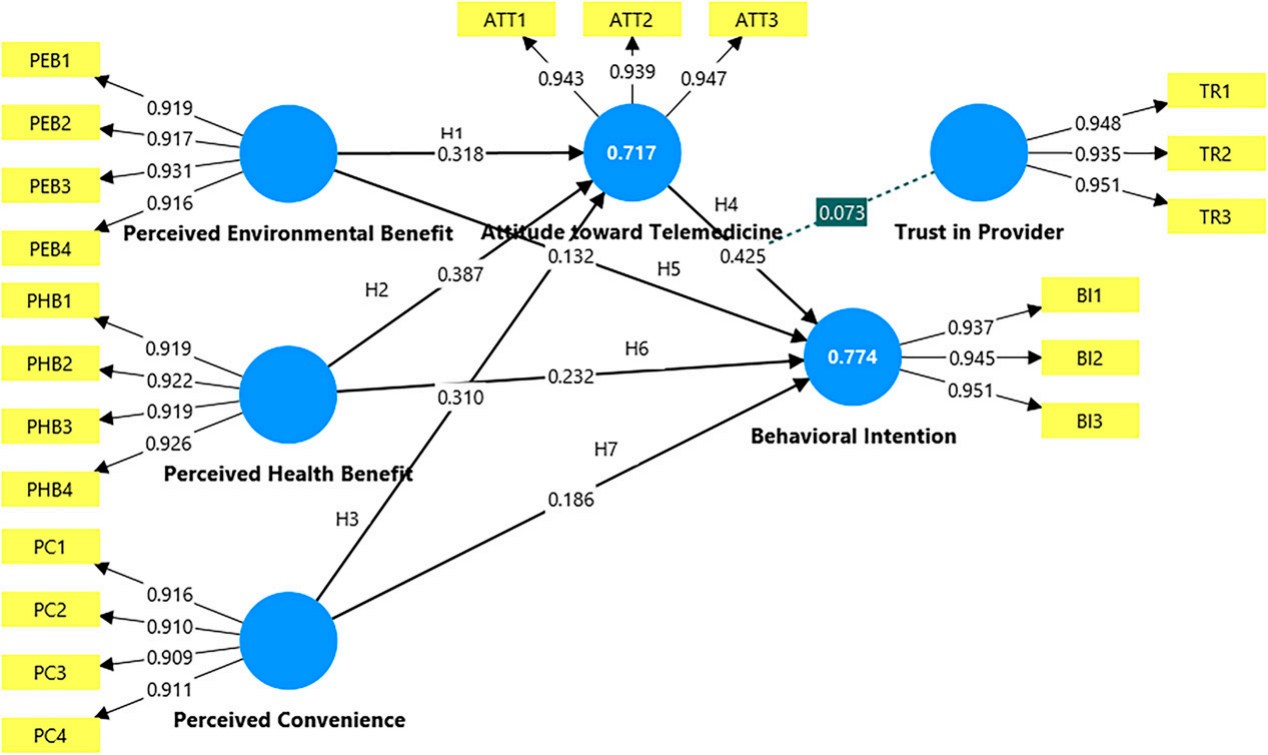
Structural model with standardized path coefficients (β), explained variances (*R*²), and moderating effect.

Figure 2 illustrates the final structural model with standardized path coefficients, *R*^2^ values and the significant moderating effect of TR.

### Model evaluation and interpretation of figures

Figure 1 presents the conceptual framework developed for this study, integrating the TPB with sustainability marketing logic. The model proposes that three belief constructs, PEB, PHB, and PC, shape ATT, which in turn influences BI to use telemedicine. TR is hypothesized to moderate the ATT–BI relationship, while ATT mediates the effects of PEB, PHB, and PC on BI. This structure reflects both the cognitive (belief-based) and relational (trust-based) processes driving sustainable telemedicine adoption.

Figure 2 shows the tested structural model and standardized path coefficients obtained through PLS-SEM (5,000 bootstrap samples). All hypothesized direct paths were statistically significant and positive. PEB (β = 0.318, *p* < 0.001), PHB (β = 0.267, *p* < 0.001), and PC (β = 0.231, *p* < 0.001) each had strong effects on ATT, explaining 69% of its variance (*R*^2^ = 0.69). ATT itself strongly predicted BI (β = 0.412, *p* < 0.001), confirming its central role in telemedicine adoption. The three benefit constructs also showed smaller but significant direct effects on BI (β values between 0.112 and 0.198, *p* < 0.05), indicating partial mediation through ATT. Bootstrap-based indirect effect testing (95% CI) confirmed that ATT significantly mediated the relationships between PEB, PHB, and PC and BI (VAF = 41.4–50.6%), suggesting that favorable attitudes act as a psychological bridge between perceived benefits and intention to adopt.

The moderating effect of TR on the ATT–BI path was positive and significant (β = 0.073, p = 0.002). Simple slope analysis showed that the effect of ATT on BI was stronger for individuals with high TRs (+1 SD) compared to those with low trust (−1 SD). This confirms that trust amplifies the translation of positive attitudes into BI, reinforcing the relational dimension of telemedicine acceptance.

Collectively, these results demonstrate that telemedicine adoption is driven by a multi-value logic, combining environmental responsibility, health utility and service convenience, and is strengthened by trust-based confidence in digital health care providers. The model’s explanatory power (*R*^2^ = 0.69 for ATT, *R*^2^ = 0.74 for BI) and predictive relevance (Q^2^ > 0) indicate a strong and well-fitting structural model, supporting the theoretical integration of TPB with sustainable marketing and digital trust mechanisms.

### Summary of findings

The measurement model demonstrates good reliability (α ≈ 0.93–.94; ρc ≈ 0.95–.96) and convergent (AVE ≥0.83) and discriminant validity (HTMT <0.90). The structural model confirms PHB as the strongest predictor of ATT and BI, followed by PEB and PC. ATT has the largest effect on BI and TR moderates the ATT→BI relationship (significant moderation). Significant *R*^2^ values (ATT = 0.69; BI = 0.74) establish strong explanatory power and mediation tests validate ATT partially mediates the influences of PEB/PHB/PC on BI.

## Discussion and Implications

### Summary of key findings

The research proves that telemedicine adoption is cumulatively affected by PEB, PHB, and PC, while ATT is the most significant intervening variable to BI. Structural analysis finds that all three advantages have a significant impact on ATT (PEB β = 0.318; PHB β = 0.387; PC β = 0.310), and ATT has a significant impact on BI (β = 0.425). All the benefits also have a significant direct impact on BI (PEB β = 0.132; PHB β = 0.232; PC β = 0.186), directing toward partial mediation by ATT. Further, TR has a positive influence on the association between ATT and BI (β = 0.073), whereas its own direct influence on BI is not substantial, reaffirming it as a moderator instead of an independent variable. The model accounts for 69% of the variance in ATT and 74% of variance in BI, reflecting high explanatory power. Bootstrapped indirect effects tests also verify significant PEB, PHB, and PC ATT-mediated paths (β = 0.135, 0.164, and 0.132, respectively; all *p* < 0.001). Together, these results strengthen that convenience, environmental, and health benefits converge to enable telemedicine intention and that trust enforces positive attitudes’ power, providing strong evidence supporting the theoretical framework suggested.

### Theoretical contributions

This study contributes theoretically by situating sustainability marketing in a digital health adoption context and by showing that PEB is not a peripheral cue but a first-order determinant of telemedicine usage. By synthesizing the TPB with sustainability and service-convenience logic, the model shows that environmental, health, and convenience beliefs collectively influence attitude and intention, thus expanding TPB beyond purely utilitarian motivations. The findings emphasize the mediating function of attitude, explaining how a variety of benefit perceptions are converted into intention to act with some partial direct effects being permitted. The research also reveals TR as an important boundary condition, illustrating that trust enhances the attitude—intention relationship and thus clarifies the comprehension of how credibility and perceived risk affect the adoption of sustainable services. By disentangling the relative salience of environmental, health and convenience advantages and by including moderation and mediation in one model, this study adds to telemedicine and sustainable marketing theory and offers a scalable model for studying other digital services with environmental co-benefits.

### Managerial and policy implications

The results offer direct implications for policymakers, telemedicine providers and health care managers who are keen to enable telemedicine as a sustainable service. Providers ought to prioritize health benefits first, given that they exert the greatest collective effect on BI, while supplementing messages with quantified environmental co-benefits like CO_2_ savings and travel avoided in order to take advantage of consumers’ increasing eco-concerns. Service design must optimize convenience, for instance, by making scheduling and multi-device access effortless, since convenience has a direct and indirect influence on intention. As TR reinforces the power of positive attitudes, organizations must indicate data privacy, security, and clinical quality at decision moments, e.g., appointment booking or consent screens. Policymakers can reinforce these firm-level efforts by creating credible eco-labeling or certification programs for telemedicine and incentivizing low-carbon care pathways, such as through reimbursement systems or public awareness campaigns. Collectively, these approaches allow health care marketers and regulators to position telemedicine as a dual-value innovation, health-enhancing and planet-friendly, while leveraging trust and convenience to translate sustainability messaging into long-term adoption.

#### Economic co-benefits and sustainability value

In addition to health and environmental co-benefits, telemedicine also produces immense economic co-benefits for health care systems and patients. Individuals also view telemedicine as a cost- and time-saving substitute for face-to-face consultation, mitigating travel, work absenteeism and facility cost charges. Perceived cost and time advantages boost overall satisfaction and fortify BI by affirming service’s dual value, environmental stewardship and cost efficiency. That appeals to prior work revealing how sustainable innovations are more accepted broadly when their benefits are not merely ecological but also affordable for their end-users.^13,14^ Therefore, framing telemedicine as an eco-efficient service, emission, money, and time-saving may expand market acceptability, assist health care cost-containment efforts, and serve sustainability goals simultaneously.

### Robustness and validity checks

They are supported by high reliability of measurement (α and ρc both more than 0.90), convergent validity (AVE ≥0.83), discriminant validity (HTMT <0.90), low collinearity (VIF <5), and satisfactory global fit (SRMR well below 0.08). Collectively, they support the stability and interpretability of the structural paths.

### Comparative and theoretical positioning

These findings are aligned with and extend previous work supporting mobile health (mHealth) and wearable-device adoption research. Both bodies of work point to perceived usefulness, convenience, and trust as primary drivers of adoption, concerns common to their emergence within the telemedicine context.^10,27^ The work here extends this prior work, however, by our incorporation of sustainability value, notably perceived environmental value, as a primary condition of intention to adopt. Whereas prior mHealth and wearable work focus on individual advantage and self-monitoring benefit, respectively, telemedicine engages a communal benefit aspect, by which patients might see involvement as an issue of, both, individual health choice and social and environmental obligation.

Positioning its results within sustainability-transition theory, the research contributes to explaining how market and social systems diffuse health care innovation aligned with environmental values. Sustainability-transition theory contends that technological transformation into sustainable procedures relies on supporting niches where innovation meets user requirements, institutional approval and societal advantage concurrently.^39^ Telemedicine is a case fitting the bill: it is a niche innovation linking digital transformation and low-carbon health care goals. Therefore, incorporation of sustainability beliefs into telemedicine diffusion models marks a move of acceptance models based on utilitarian grounds to value-based diffusion models, linking digital transformation and ecological modernization of health care.

### Limitations and future research

While this research provides strong support for the hypothesized model, a number of limitations indicate avenues for further research. First, causal inference is precluded by the cross-sectional design; future research may use longitudinal or experimental designs to test change over time in perceptions and intentions and to experiment with the long-term effect of sustainability messaging. Second, data are based on self-report measures, which can be vulnerable to social desirability or recall bias; supplementation with surveys to objectively use data or passive digital traces would increase validity. Third, the research was carried out in one national and service setting, which could restrict generalizability; replications in various health care systems and cultures could challenge boundary conditions such as insurance coverage, infrastructure maturity, and environmental norms. Fourth, the model is centered on general benefit perceptions and trust; additional constructs can be added in future research, e.g., perceived cost savings, technology readiness, or environmental identity, to increase explanatory power. Last, experimental message-framing investigations (e.g., health-first vs. environment-first framing, numeric CO_2_ feedback vs. qualitative impact) might uncover how communication strategy differences and individual differences (eco-concern, digital literacy) moderate present effects. Addressing these limitations will serve to validate and broaden the broader generalizability of the current findings and theoretical and practical conceptions of sustainable telemedicine marketing.

## Conclusion

This research aimed to clarify how perceived environmental, health, and convenience benefits influence ATT and eventually BI and investigate the moderating effect of TR and the mediating effect of attitude. With the Theory of Planned Behavior and sustainability marketing theory as our guide, we conducted a survey among 400 adult consumers and used PLS-SEM to analyze the data. The model was of satisfactory measurement quality and possessed good explanatory value (*R*^2^ = 0.69 for attitude; *R*^2^ = 0.74 for intention to exhibit the behavior). Major findings affirm that perceived environmental, health, and convenience advantages all positively influence ATT, which then influence intention. Each advantage has a direct influence on intention, showing partial mediation.

Moreover, the provider’s trust significantly enhances the role of attitude over intention, further highlighting the credibility and perceived risk reduction values in telehealth adoption.

Theoretically, the study incorporates sustainability value into digital health uptake models and makes attitude a central mechanism by which various benefit perceptions are translated to BI. It also forms the basis that technologies are a desirable boundary condition, adding both technologies and health-service as well as marketing technologies literature. Practically, the study demonstrates that telemedicine providers can encourage stronger consumer uptake when they introduce and report environmental co-benefits quantitatively, provide health outcomes, are convenient in design and provide privacy and security guarantees.

As with any research, this research also contains its own set of limitations, its cross-sectional nature, use of self-report data, and single national context setting, that encourage future research using longitudinal, experimental, or cross-cultural designs. Future research can also test cost perceptions, digital literacy or environmental identity as alternative explanation variables and examine alternative message framings (e.g., health-first vs. environment-first) and platform design signals promoting sustainable behavior.

Briefly, the evidence shows that telemedicine is less a pandemic-born or convenient care innovation and more of a twin-value solution to increase health care access and environmental stewardship. By framing telemedicine as an option for people-healing and planet-preservation and through establishing patient trust, health care organizations and policy leaders can translate positive consumer perceptions into long-term, widespread adoption, delivering better health outcomes while helping meet global climate objectives.

## Abbreviations Used

AVE: Average variance extracted
BA: Brand accessibility
CFA: Confirmatory factor Analysis
CR: Composite reliability
PBT: Perceived Brand Trust
PE: Perceived empathy
SEM: Structural equation modeling
SD: Standard deviation
SOR: Stimulus–organism–response
